# Supplementary figures and images for: Input Zone-Selective Dysrhythmia in Motor Thalamus after Dopamine Depletion
Source: J Neurosci. 2021 Dec 15;41(50):10382–404. doi: 10.1523/JNEUROSCI.1753-21.2021 (PMC8672689; doi:10.1523/JNEUROSCI.1753-21.2021)

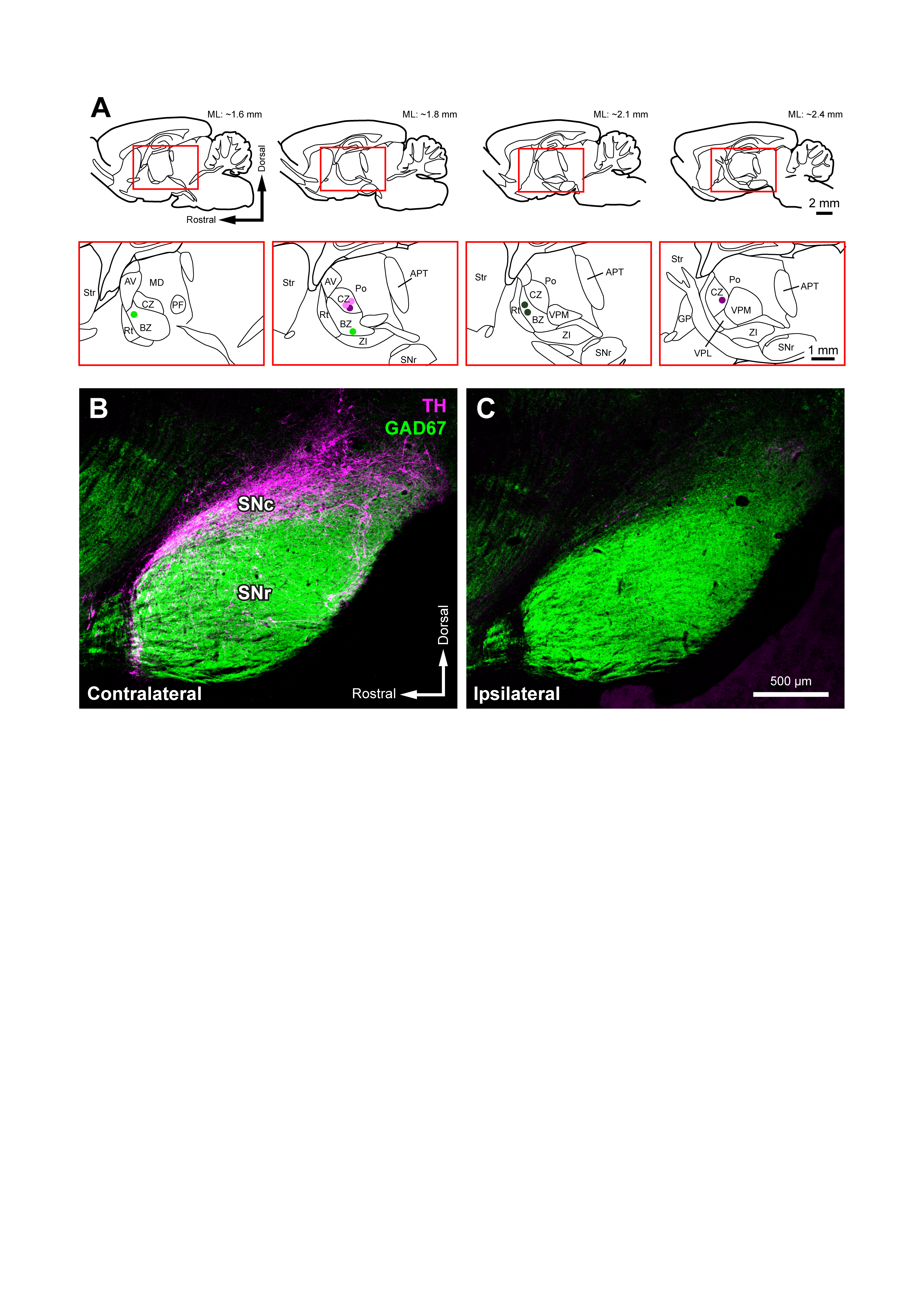

Supplement: Figure 1-1 — Locations of neurons in the motor thalamus of rats, and anatomical verification of 6-OHDA lesions. A, Locations of the BZ and CZ of the motor thalamus as mapped onto larger parasagittal sections of the rat brain for additional anatomical context. ML is distance lateral of bregma. The red boxed areas in sections of upper row are expanded in the lower row. Locations of the recorded and identified neurons shown in Figure 1 are highlighted; BZ neurons recorded in dopamine-intact and 6-OHDA-lesioned rats are marked by light green and dark green dots, whereas CZ neurons recorded in dopamine-intact and lesioned rats are marked by pink and purple dots, respectively. APT, anterior pretectal nucleus; AV, anteroventral thalamic nucleus; GP, external globus pallidus; MD, mediodorsal thalamic nucleus; PF, parafascicular thalamic nucleus; Po, posterior nuclear group of thalamus; Rt, thalamic reticular nucleus; SNr, substantial nigra pars reticulata; Str, striatum; VPL, ventral posterolateral thalamic nucleus; VPM, ventral posteromedial thalamic nucleus; ZI, zona incerta. B, C, Images of parasagittal tissue sections of the midbrain contralateral (B) and ipsilateral (C) to the site of a 6-OHDA injection made in the same rat. Both sections were processed for immunoreactivity for tyrosine hydroxylase (TH) to reveal dopaminergic neurons in the substantia nigra pars compacta (SNc), as well as for immunoreactivity for glutamic acid decarboxylase of 67 kDa (GAD67) to delineate the SNr. Note the profound reduction in the prevalence of dopaminergic neurons in and around the SNc ipsilateral to the 6-OHDA injection. Download Figure 1-1, TIF file. [file ns-JN-RM-1753-21-s01.tif]

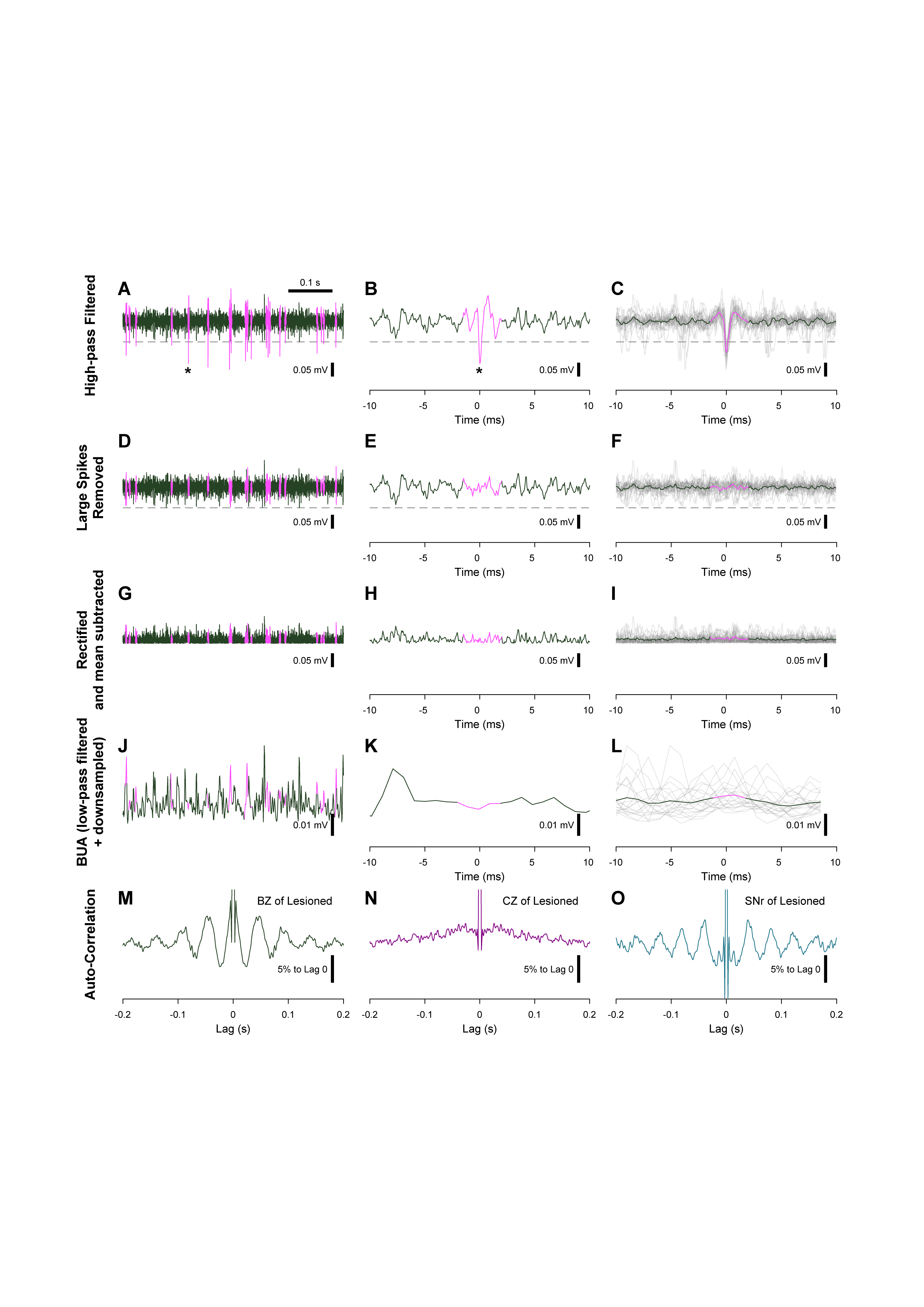

Supplement: Figure 6-1 — Steps for extraction and processing of BUA signals for time-series analyses. Wideband recordings made with silicon probes were high-pass filtered at 300 Hz, and then any large-amplitude action potentials (spikes) were detected (A-C). Pink spikes in A are those identified as being of large amplitude (crossing the threshold of 3 standard deviations indicated by the dashed line), with asterisks in A and B indicating the same large spike. These large spikes were then removed and replaced with another randomly selected part of the recording that did not contain large spikes (D-F). Replacement data are also highlighted in pink in D-L. The resultant BUA signals were then rectified and mean subtracted (G-I) before being low-pass filtered at 300 Hz and downsampled to 1024 Hz to generate a continuous measure for further analyses (J-L). Portions of individual signals at each processing step are shown at low (A, D, G, J) and high (B, E, H, K) temporal resolutions. Several traces are overlaid in relation to the detected large-amplitude spikes to clarify the effects of removing spikes and other signal processing steps (C, F, I, L). M-O, Examples of autocorrelation functions of processed BUA signals recorded in the BZ (M) and CZ (N) of the motor thalamus and in the SNr (O) of 6-OHDA-lesioned rats. Note in the BZ and SNr autocorrelations the presence of multiple peaks every 40-50 ms, reflecting oscillations in the beta-frequency band (15-30 Hz). Download Figure 6-1, TIF file. [file ns-JN-RM-1753-21-s02.tif]
